# Impact of different rectangular wires on torsional expression of different sizes of buccal tube

**DOI:** 10.4317/jced.54300

**Published:** 2018-01-01

**Authors:** Shabnam Ajami, Afshar-Rasti Boroujeni

**Affiliations:** 1 Assistant Professor, Orthodontic Research Center, Department of Orthodontics, School of Dentistry, Shiraz University of Medical Science, Shiraz, Iran; 2 Undergraduate Student, Student Research Committee, School of Dentistry, Shiraz University of Medical Science, Shiraz, Iran

## Abstract

**Background:**

Torsions in rectangular wires are the essential part of corrections in the finishing stage of treatment. Moreover the greatest amounts of torques are applied in the molar areas. a clinically effective moment is between 5 and 20 Nmm. In this study we have decided to evaluate the impact of different tube sizes and different dimensions of wires with different modulus of elasticities on the amount torsional bond strength of molar tubes.

**Material and Methods:**

60 human impacted molar teeth were collected. A buccal tube was bonded on the buccal surface of all the samples by using light cured adhesive resin. After that, the teeth were mounted in a hard acrylic block. According to the size of buccal tube and the rectangular wires to be tested 4 groups will be designed. Torsional force was applied by instron machine. The torque angle at 5Nmm and at 20Nmm point will be calculated: which means, how many degrees of torque is required to reach the maximum 20Nmm moment from the minimum 5Nmm.One-way ANOVA was used to compare torque angle in all of the groups.

**Results:**

The least amount of clinically significant angle was 2.2 ᵒ in the 0.017×0.025 SS and the largest amount of it was 23.7 ᵒ in the 0.017×0.025 TMA in 0.018×0.025 slot molar tube. But, this angle was 19.9 ᵒand 13.6 ᵒ in 0.019×0.025 SS and 0.019×0.025 TMA archwire in 0.022×0.028 molar tube.

**Conclusions:**

The 0.017×0.025 SS archwire in 0.018×0.025 molar tube had the lowest clinically significant angle. The largest amount was seen in group 0.017×0.025 TMA in 0.018×0.025 slot molar tube.

** Key words:** Torsional efficacy, rectangular wires, buccal tubes, torque angle.

## Introduction

To have a correct axial inclination at the final stages of fixed orthodontic treatment, a controlled root movement is required which is reffered to as third order movement. A twist of an edgewise wire in any attachment slot provided the required torsional load for root up righting (1).

The couple force provides rotation of teeth in bucco-lingual direction is called torque. Torque can be observed from a mechanical or clinical point of view. Mechanically, it is the rotation of structure around its long axis. Clinically, it is the bucco-lingual crown/root inclination. This would be the any rotation perpendicular to the long axis (2).

As defined by preview studies, for the purpose of the study torque is defined as the physical couple force applied to the molar tube, measured in Nmm.torque angle is considered the angle to which the wire is twisted (degree).and torque axpression is the torque at any set of angles (3-5).

Torque expression is the result of many interacting factors. Attachment designs, engagement angle of wire and slot, mode of ligation (6), attachment deformation, mechanical properties of wire (6,7), wire edge beveling (8-10), are declaredas factors impacting the torque expression. Most fixed orthodontic treatments are accomplished with less than full-dimention wires. This means there is a lack of cohesive contact between the archwire and attachment when an undersized wire is inserted, the wire can rotate in slot of attachment .

This angle of freedom is called play and it would increase as the differences in size between the slot and wire (11).

In the range of this play no third-order movement would happen. In 1892, Burston defined the clinically effective movement between 5 and 20 Nmm which means no root movement happens under 5 Nmm and exceeding 20 Nmm may deteriorate the predontium (12).

This effective sizeof the slot is the most important factor influencing the biomechanics during orthodontic treatment. The appliances introduced by angle possessed the slot height was 0.022 inch, however by introduction of smaller archwires attachments with slot 0.018 inch get popular. This slot size was threaded with working archwires of 0.017× 0.025 and 0.018 × 0.025 as full-thickness archwires (13). Thereafter, Roth reintroduced slot size of 0.022 with the straight wire technique (14). At the same, the archwires start to evolve, followed by introduction of beta-titanium (TMA) atchwire by the Ormco Corporation with the elasticity between steel and NiTi (15).

As stated torsions in rectangular wires are the essential parts of corrections in the finishing stage of treatment. Moreover the greatest amounts of torques are applied in the molar areas. As the result, in this study we have decided to evaluate the impact of different tube sizes and different dimensions of wires with different modulus of elasticities on the amount torsional bond strength of molar tubes. Also the maximum torque that can be applied prior to debonding of buccal tube will be determined.

## Material and Methods

-Specimen preparation:

At first 60 third impacted molars which were extracted due to space deficiency diagnosed in the posterior segment analysis of mandibular arch were collected. All specimens were evaluated to have an intact buccal surface and not having any form of cracks, fracture, void or enamel developmental defects. They were stored in o/1 % Thymol solution to control bacterial growth. Before bonding, buccal surfaces of all teeth were cleaned with pumice and rinsed with distilled water.

A buccal tube (American Orthodontic, USA) was bonded on the buccal surface of all the samples under controlled temperature (37º) and humidity (54%+_5): relative humidity, using the following procedures as recommended by the manufactures guidelines. The buccal enamel surface in the middle third of occlusogingival and mesiodistal dimension was etched for 30 seconded using phosphoric acid (3M, Monrovia, USA) and rinsed for 20 seconds. After that, the etched enamel was dried with oil and moisture free compressed air. Then Tansbonded XT primer (3M, Monrovia, USA) was applied using a microbrush. Tubes were bonded using light cured adhesive resin Transbond XT 3M, Monrovia, USA. The base of buccal tube was coated a uniform layer of Transbond XT light cure adhesive resin. To position all the tubes in the same position on the buccal surfaces, excess of resins were removed with a dental explorer, an orthodontic gauge was used. Position of the buccal tubes was reexamined and each margin of tube bases was cured for 20 seconds with an Optilux visible light curing unit (Kerr corp, Orange, Calif, USA). Before each bonding, the curing light was tested with a curing radiometer (Kerr, corp, Orange, Calif, USA) for a minimum intensity at 400 mw/cm2.

After bonding of buccal tubes, the teeth was mounted in a hard acrylic block (Pars Acryle,Iran). For mounting a putty index was prepared and the teeth was placed in acrylic blocks using a surveyor (Marathon, South Korea) in a way that the buccal tubes were positioned at the right angle to horizon.

This omitted any pre adjusted torque in the buccal tubes which can be caused by malposition of teeth in the acrylic blocks. According to the size of buccal tube and the rectangular wires to be tested 4 groups were designed.

Group 1: buccal tube size of 0/018 × 0/025 inch and the tested wires of 0/017 × 0/025 inch SS.

Group2: buccal tube size of 0/018 × 0/025 inch and the tested wires of 0/017 × 0/025 inch TMA.

Group3: buccal tube size of 0/022 × 0/028 inch and the inserted wire of 0/019 × 0/025 inch SS.

Group4: buccal tube size of 0/022 × 0/028 inch and the inserted wire size will be 0/019 × 0/025 inch TMA.

-Testing apparatus:

A testing apparatus was designed to fix the orientation of the buccal tubes to the rectangular arch wires in all three planes. The apparatus mimicked rectangular arch wire torqueing. A holder was considered at the middle of this apparatus to hold the specimens blocks.

Supporting posts were constructed to mount a cross bar so that the wire could twist without any displacement in other directions. A string was tied around a drum on the torque axis on one side of crossbars. The other end of the string was fastened to a 100-N load cell in the universal. Testing machine (Zwick/Roell, Z020, Germany)the speed of crosshead was 0.5 mm/min (16,17) the wire was inserted in the buccal tube at each sample and grasped on both sides by crossbars. The wires were aligned with the buccal tubes so that no existing torque or angulation existed. For each sample displacement (mm) at 5Nmm and at 20Nmm of moment was recorded by the instron machine (Zwick/Roell, Z020, Germany) (4).

The torque angle at 5Nmm and at 20Nmm point was calculated by converting the linear displacement of the string (in millimeter) which was needed to twist the wire united to rotational motion (in degree) and for each single sample in different combination of wires and tube size, the clinical significant torque angle was calculated as follow: which means , how many degrees of torque was required to reach the maximum 20Nmm moment from the minimum 5Nmm.This torque was expressed in either clockwise or counter clockwise.

## Results

The mean and standard deviation (SD) of the angles at different combination of the archwires and tube slot sizes showed the couple of 5 and 20 Nmm, demonstrate in  Table 1.

**Table 1:**
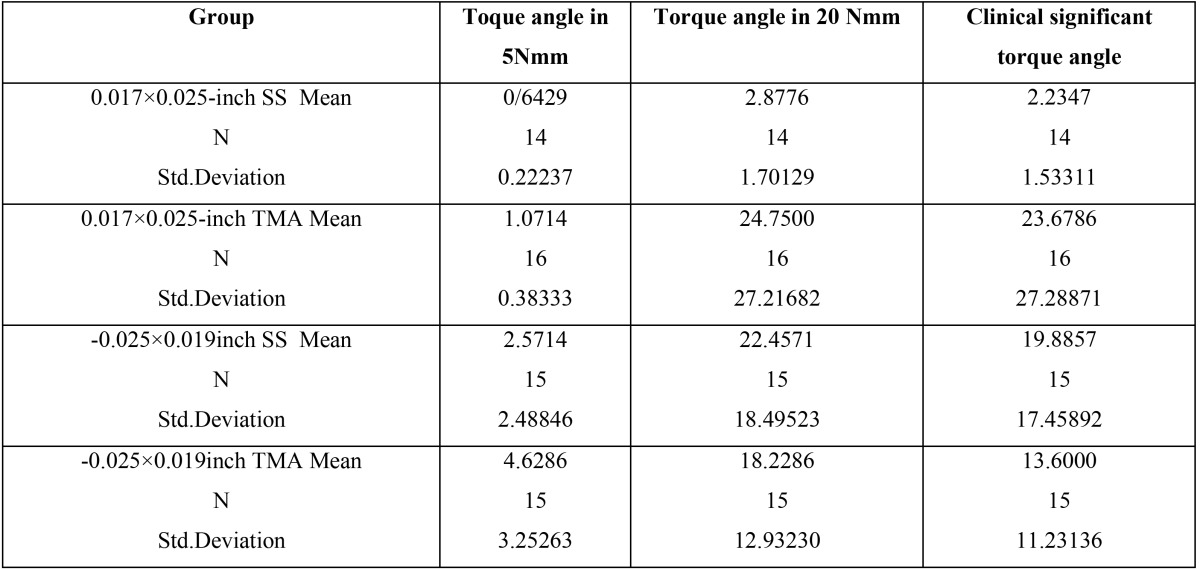
The mean and standard deviation (SD) of torque angle in 5Nmm, 20 Nmm and clinical significant couple.

The final column of the table demonstrates the clinical significant couple interval for each combination. This means the amount of torque needed to be appeared to reach the maximum 20Nmm couple from the minimum 5Nmm, either in clockwise or counterclockwise. Comparison of different combination of tube sizes and archwires showed a clinically significant difference in the amount of torque angle needed to reach 5 Nmm, 20Nmm and also the amount of clinically significant torque angle among the groups. The least amount of clinically significant angle is shown in the combination 0.017×0.025-inch SS archwire in 0.018×0.025-inch molar tube which was 2.2 ᵒ.

In comparison between each two groups, this combination (0.017×0.025-inch SS archwire in 0.018×0.025-inch molar tube) was significantly lower than other groups.(*P*<0/001)( Table 2)

**Table 2:**
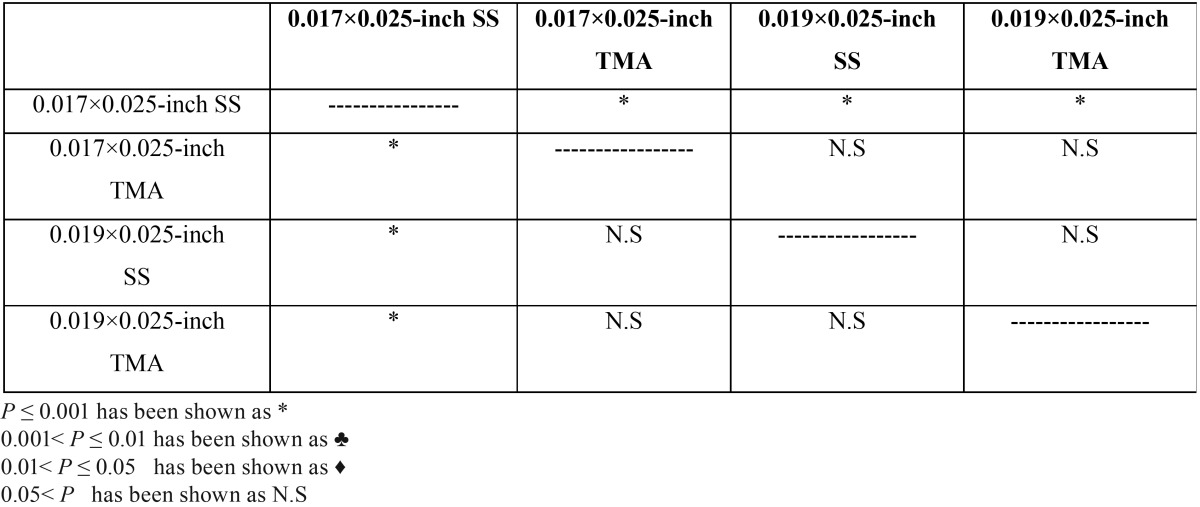
Comparison between groups based on clinical significant couple.

Although the largest amount of couple at 20 Nmm which is the threshold of clinically acceptable one was seen in the 0.017×0.025-inch TMA archwire in 0.018×0.025-inchslot in molar tubes, it was not statistically significant in comparison with the other two groups of 0.019×0.025-inch archwire in slot size of 0.022×0.028-inch with different material properties (*P*>0/05).

**Figure 1:**
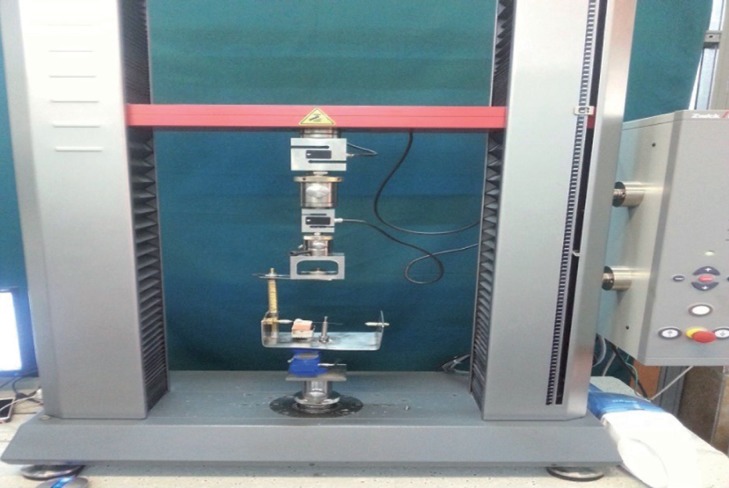
Instron machine (Zwick/Roell, Z020, Germany), The constructed testing apparatus mounted on the machine.

**Figure 2:**
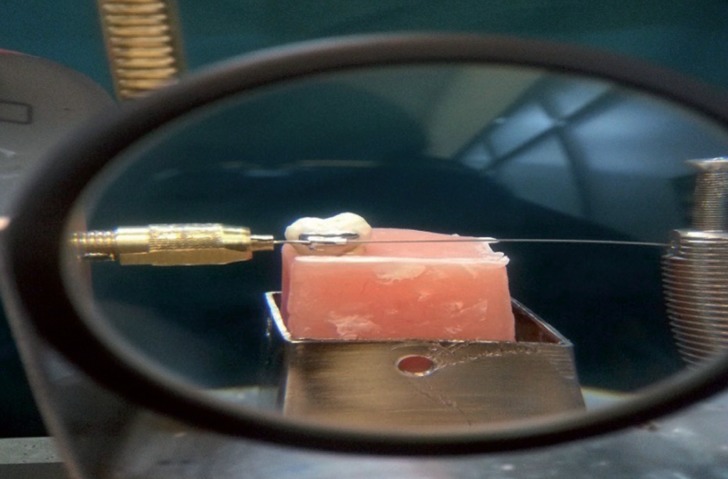
A sample in the acrylic block with a bonded molar tube grasped in the apparatus and a torsional load is applied by the wire inserted in the tube.

The largest clinically significant torque was also reported in group0.017×0.025-inch TMA in 0.018×0.025-inch slot molar tube ( Table 2- Table 4, Figs. 1,2).

**Table 3:**
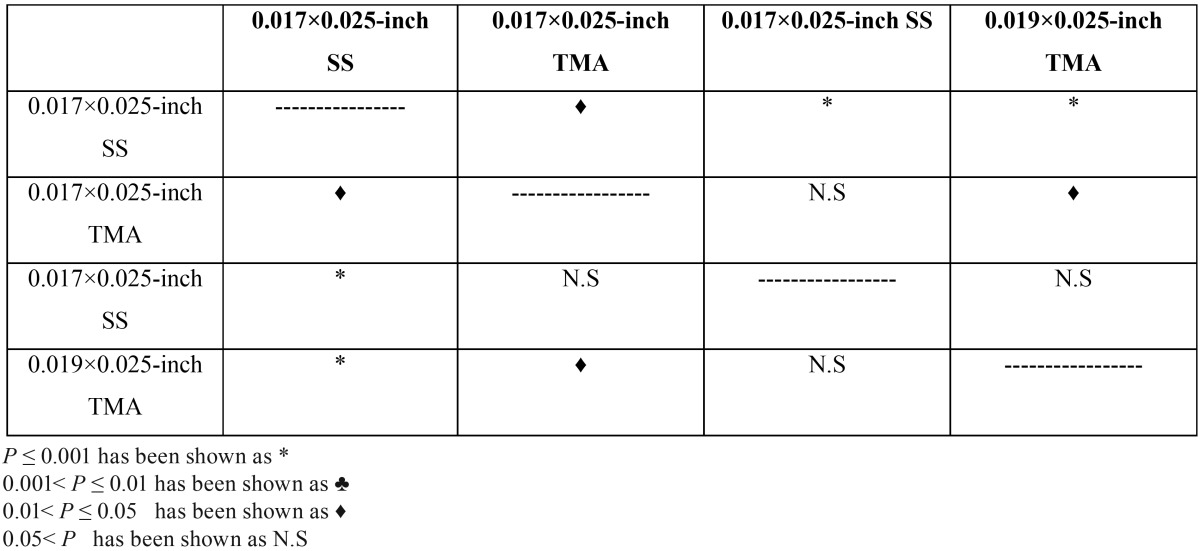
Comparison between groups at 5Nmm torque.

**Table 4:**
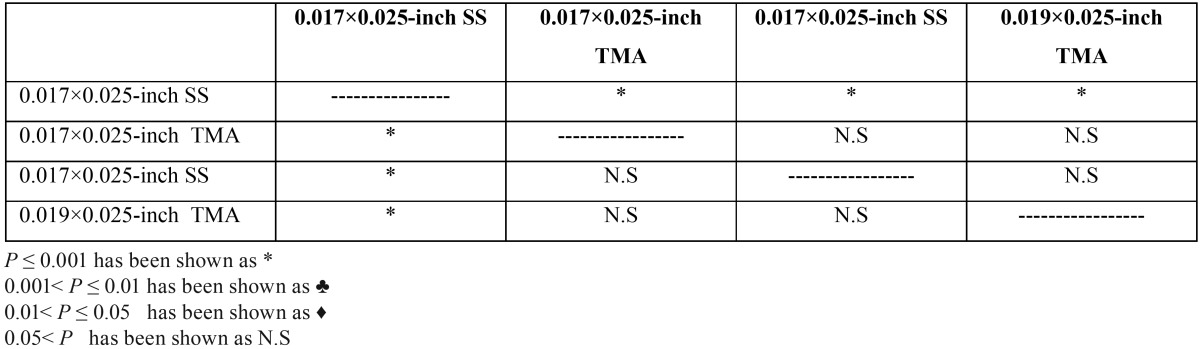
Comparison between groups at 20Nmm torque.

## Discussion

In the clinical practice during the finishing stage of fixed orthodontic treatment mostly the evaluated configurations would be used. Heavier archwires such as 0.021×0.025 –inch are rarely used in practice due to generated hazardous forces regarding root resorptions (18). The teeth which were under loads of moments with higher magnitude for a longer duration are predisposed to a higher degree of root resorption.

Although, the maximal torque threshold is hard to defined, Burston defined the 20Nmm moment at the maximal limit over which is likely to damage the periodontal tissue (12). Moreover they determined the range between 5 Nmm and 20 Nmm as the clinically efficacious moments.

The etiology of root resorption is multifactorial and mechanical factors cannot extrapolate all this clinical situations.

Even at any specific moment magnitude, the location of center of rotation influence the pressure area on the periodontium. It also should be put into consideration the fact that the relationship between center of rotation and center resistance may change over time which is a rule rather than an exception.

By knowing the above fact, the difference in clinically significant torque angle detected among different configuration are caused by the differences in wire material and cross sections. The clinically significant torque angle of 0.017×0.025 SS archwire in 0.018 inch system tube had the lowest degree. This low torque angle is almost impractical to be applied precisely in clinic. This result consistent with the result reported by Sifakakis *et al.* that the greatest mean moment of 14.2 Nmm was reported in 0.017×0.025 SS archwire in the 0.018-inch brackets in 15ᵒ buccal root torque (19).

The elastic properties of 0.019×0.025-inch TMA are identical to an 0.018-inch SS wire and almost have half stiffness of steel wires of the same cross sections.

Both play and applied torque are affected by the attachment and archwire features. Though the material used to construct the archwire is not an impacting factor for the amount of play, it is an important factor in term of applied torque. For bracket type attachments, Archambault et al reported that at any angle of torque, 0.019×0.025 SS archwire expresses a couple 1.5 to 2 times greater than TMA (5).

For the molar tubes with the twist of wire from the mesial side, as was tested in the present study, the among of clinically significant torque angle was 1.5 times in SS in comparison to TMA in 0.022-inch system .On the other hand with less play in configuration of 0.017×0.025 in 0.018-inch molar tube, this ratio increased to more than 10times.

Moreover, in a recent investigation by Mager et al, the plastic deformation of the sloth is negligible in the couples less than 26 to 38 Nmm (18).

In the present study, the moment never exceeded 20Nmm. The plastic deformation of metal walls could not deteriorate the results. Since, the wearing of interior walls of tubes due to numerous times of testing could influence the result (20,21). For each configuration, a new archwire and molar tube was assembled in this study.

The differential effect of the interbracket distance was almost omitted because the length crosshead was identical in all samples, and it was assumed to be as close as possible to clinical situation. On the other hand there are no significant effects of length on torsion and changes in length are not an exponential factor as they are in bending (22).

In previous studies on this subject, the torque expression were evaluated in bracket slots and the type of ligation of the wire in the slot could have an impact on the results (12). In the present study the torque efficacy of different wires have been evaluated in the molar tubes. Since there is only insertion and no ligation this factor has been eliminated, and this configuration has been chosen is because of the importance of torque on posterior segment and there were no previous study on this this subject.

It was found that after elimination of the play, the torque moment was significantly higher in wire ligation than elastic ligation in torsion levels of 40˚. However these differences were not observed in fully engaged archwires.

The indisputable drawback of elastic ligation is the rapid force degradation which could be 50% in the first 24 hours which could lead to incomplete engagement of the wire in the slot.

In preadjusted systems, the typical torque prescription is less than 25 degrees for molar tube and the use of straight wires in 0.022-inch systems TMA and SS with the size of 0.019×0.025-inch in the finishing stage would be in the limit of acceptable couple forces with regard to peridontiom.

For 0.018-inch systems, the 0.017×0.025-inch ″ SS would exceed the safe zone after 2˚ of torque. In molar tube this force would remain till the tooth movement accurse and no degradation due to elastic ligation is present, an in preadjusted systems with prescribed torque which mostly exceed 2˚ of torque in molar areas, insertion of 0.017×0.025 ″-inch SS and heavier SS wires in molar tubes could be damaging (3).

Although, TMA wire with their reduced torque angular deflection ratio in comparison to SS wire are less effective in transmitting the desire torque moments to the attachments, their range of possible activation is more fail safe in comparison to SS wires specially in 0.018-inch systems.

In comparison of previous studies on torque expression different configuration of brackets including various type of self-ligating brackets are compared with conventional one (19).

In these studies the mechanical properties of the materials used to construct the attachments, such as modulus of elasticity and roughness of the slot walls arises impact on the amount of torque expression (19).

In this study, this factor was eliminated by using the same brand in all samples and only the types of wire were the investigated factors.

However, it is proposed to investigate these properties as the influencing factors in the future studies on this subject.

Another important factor in the clinical routine situation is the vertical position of the attachments, since as previously was demonstrated a 3mm shift in vertical position can change the torque angle about 15 degree (23).

This factor has less impact in molar tubes due to the fact that most of the times at least one side of the tube is the free end of the wire in comparison to the brackets attached to other teeth in fixed orthodontic systems.

This element can also be investigated on the future studies especially since the finishing steps using TMA rectangulatr wire could happen often.

The fact that a 10 degree less in the amount of torque may cancel out the prescribed torque in preadjusted attachment of the produced torque by this elements is in the opposite direction (6).

The present study focused on the loading expression as many previous studies. With considering this fact that the unloading torque characteristics result in the root movement, we propose that these characteristic also be investigated in the future studies.

It is important to consider that many factors are involved in the final inclination and position of teeth in the finishing stages of orthodontic treatments.

Torsion magnitude is only one factor from many mechanical properties; such as: thickness of wire, position of bracket and tooth, slot sizes, attachments composition, the manufacturing tolerance and processes of wire attachments, as well as intra oral aging (24,25).

This study is only a simplified representation of what occurs in the oral cavity.

In addition to all these mechanical factors, position of center of resistance, center of rotation, root length, alveolar bone height can dictate the final inclination of a tooth.

## Conclusions

As the conclusion, the combination of 0.017×0.025-inch SS archwire in 0.018×0.025-inch molar tube had the lowest clinically significant angle between 5 Nmm to 20Nmm applied torsional force, among four groups. This low torque angle is almost impractical to be applied precisely in clinic. However, the largest amount of clinically significant torque was also seen in group 0.017×0.025-inch TMA in 0.18×0.025-inch slot molar tube. But, there was no significant difference among with the other two groups of 0.019×0.025-inch arch wire in slot size of 0.022×0.028-inch with different material properties.
